# Thermodynamic Aspects in Non-Ideal Metal Membranes for Hydrogen Purification

**DOI:** 10.3390/membranes8030082

**Published:** 2018-09-16

**Authors:** Stefano Bellini, Yu Sun, Fausto Gallucci, Alessio Caravella

**Affiliations:** 1 Department of Environmental and Chemical Engineering (DIATIC), University of Calabria, Via P. Bucci, Cubo 44A, 87036 Rende, Italy; stefanobellini89@gmail.com; 2 Institute for International Collaboration, Hokkaido University, Sapporo, Hokkaido 060-0815, Japan; sunyusunyu1983@gmail.com; 3 Department of Chemistry, Faculty of Science, Hokkaido University, N10W8, Kita-ku, Sapporo, Hokkaido 060-0810, Japan; 4 Inorganic Membranes and Membrane Reactors (SIR), Sustainable Process Engineering (SPE), Department of Chemical Engineering and Chemistry, Eindhoven University of Technology, P.O. Box 513, STW 1.45, 5600 MB Eindhoven, The Netherlands

**Keywords:** chemical potential, activity, hydrides, solubility, membranes

## Abstract

In this paper, an overview on thermodynamic aspects related to hydrogen-metal systems in non-ideal conditions is provided, aiming at systematically merging and analyzing information achieved from several different studies present in the open literature. In particular, the relationships among inner morphology, dissolved hydrogen and internal stresses are discussed in detail, putting in evidence the conformation complexity and the various types of dislocations induced by the presence of H-atoms in the lattice. Specifically, it is highlighted that the octahedral sites are preferentially occupied in the FCC metals (such as palladium), whereas tetrahedral sites are more energetically favored in BCC-structured ones (such as vanadium). These characteristics are shown to lead to a different macroscopic behavior of the two classes of metals, especially in terms of solubility and mechanical failure due to the consequent induced stresses. Furthermore, starting from the expression of the chemical potential generally presented in the literature, a new convenient expression of the activity of the H-atoms dissolved into the metal lattice as a function of the H-concentration is achieved. Such an activity expression is then used in the dissolution equilibrium relationship, which is shown to be the overall result of two different phenomena: (i) dissociative adsorption of molecular hydrogen onto the surface; and (ii) atomic hydrogen dissolution from the surface to the metal bulk. In this way, the obtained expression for equilibrium allows a method to calculate the equilibrium composition in non-ideal conditions (high pressure), which are of interest for real industrial applications.

## 1. Introduction

Separation processes based on metal membranes with high hydrogen permeance and selectivity have been identified as a promising technology for efficiency improvement and cost reduction of hydrogen production. The virtually infinite selectivity of metal membranes towards hydrogen is related to the fact that the potential field of the (Pd) metal surface can dissociate only hydrogen among all the other light and heavier gas species. In particular, membranes composed of palladium and its alloys can play a fundamental role thanks to their unique affinity towards hydrogen, as they show high catalytic activity to the hydrogen molecule dissociation combined with the relatively high H-atoms diffusion [1].

The substantial difference between palladium and other metals is that the former shows a relatively small activation barrier (fast sorption kinetics) of the surface dissociation, while in the latter the activation energy of the surface dissociation is higher [2].

During the past two decades, the research on Pd-alloy membranes has led to a technology maturity that appears ready for up-scaling for applications at operating temperatures less than about 550 °C and in conditions where detrimental impurities are preventively eliminated by means of appropriate treatments (such as H_2_S removal steps).

Based on the behavior of hydrogen dissolution into the metal lattice, metals can be classified into materials in which solubility is favored by temperature and materials showing the opposite trend. The former group, including palladium and vanadium, absorbs hydrogen by means of an exothermic process generating a metal-hydride phase, whereas the latter (e.g., Pt, Fe, Cu, Ni, Ag) absorbs hydrogen endothermically forming a covalent hydride phase [3].

In the fabrication of membranes and hydrogen-storage material, it is important to limit the hydrogen solubility to avoid embrittlement and mechanical failure. For this purpose, the main metal is alloyed with others such as Cr, Co and Ni [4]. Vanadium in particular has interesting characteristics, showing the highest diffusivity and lowest solubility compared with other BCC metals [5,6,7] along with a relatively low cost. Moreover, the insertion of Ni-atoms into the V-lattice has been demonstrated to enhance the alloy properties in terms of mechanical resistance to the internal stress induced by the presence of hydrogen [8].

Current research on metal membranes has been looking for the best combination of materials and manufacturing approaches. However, such a kind of research risks to turn into a trial-and-error activity if it is not driven by the knowledge of the phenomena involved in the hydrogen-metal system. For this purpose, the present paper aims at providing a critical review of thermodynamics related to metal-H systems, giving general guidelines to improve the performances of metal membranes.

## 2. Hydrogen Interactions in Transition Metals

### 2.1. Hydrogen Dissolution in Metal Lattices

Hydrogen absorbed in metals occupies interstitial positions in metal lattices, as demonstrated by neutron diffraction [9]. The FCC lattice (like that of palladium) features an atom at each corner of the cubic cell, and one atom at the center of each face. It shows one octahedral (O) and two tetrahedral (T) interstitial sites per metal atom (14 sites and 14 metal atoms in a single cell).

**Figure 1:**
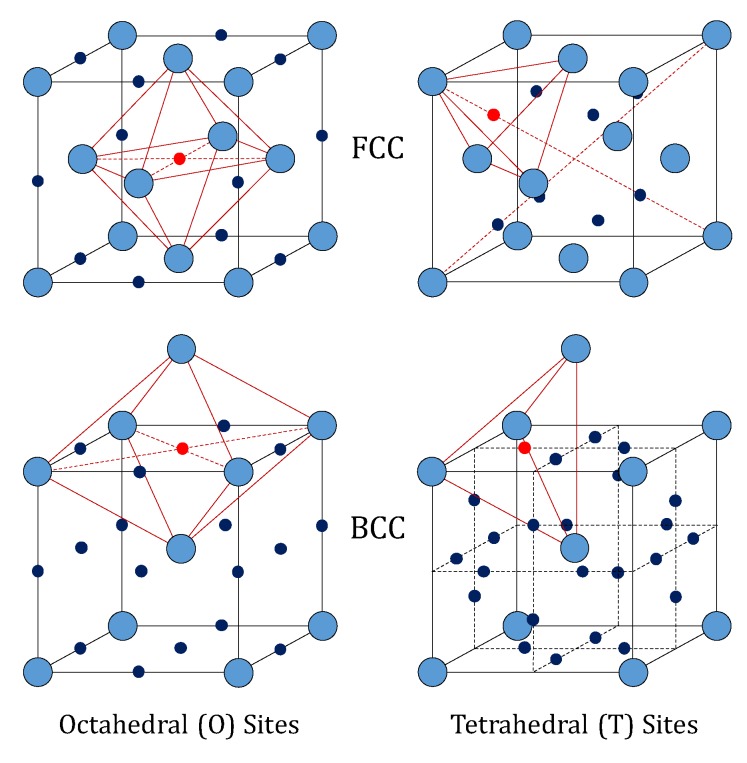
Octahedral (O) and Tetrahedral (T) sites in FCC lattice and BCC lattice [13].

Differently, the BCC lattice (like that of vanadium, niobium and tantalum) features an atom at each corner of the cubic cell and one at the center. Such a configuration has three octahedral and six tetrahedral interstitial sites per metal atom. The structures of these two lattices are depicted in Figure 1. In particular, the octahedral sites of the FCC lattice have the largest free volume, whereas the tetrahedral sites are the largest in the BCC lattice [10]. This means that the occupation of tetrahedral interstices by hydrogen in BCC lattices of the groups IV and V is favored over the octahedral ones, whereas the opposite behavior is found for hydrogen in palladium [11].

In fact, Cser et al. [12] found by neutron holographic study that the H_2_ molecule dissociates in H-atom in palladium and these atoms preferentially occupy the octahedral sites of the FCC lattice.

As for the dissolution of hydrogen in vanadium, the V-H system presents a phase diagram consisting of several different phases: (i) the solid solution (*α*-phase), observed at high temperature, in which the H-atoms are randomly distributed in the tetrahedral sites of its BCC structure; (ii) the hydride V_2_H *β*_1_-phase, showing a monoclinic structure where the H-atoms are located in the octahedral sites; (iii) the hydride V_2_H or VH *β*_2_-phase, showing a monoclinic body-centered tetragonal (BCT) structure; (iv) the V_3_H_2_ phase with a monoclinic structure; (v) the VH_2_ *δ*-phase, showing a CaF_2_-like structure with the H-atoms occupying the tetrahedral sites; (vi) the *γ*-phase VH_x_, observed at high H-concentration and temperature below 375 K ca. [13,14,15,16,17,18,19]. The overall result of such a complex situation is that neutron diffraction on vanadium-deuterium system at 50% of V-D atomic ratio showed that about 90% of the dissolved deuterium atoms occupies the tetrahedral BCC sites, with the rest being placed in the octahedral ones [20]. More recently, using a Density Functional Theory (DFT) analysis applied to pure vanadium, Lu et al. [21] estimated that the H_2_ solution energy into the T-sites is −0.332 eV, which is higher than the value calculated into the O-ones (−0.149 eV). Since a more favorable solution energy has a more negative value, this result demonstrates that hydrogen is preferentially absorbed into the BCC T-sites with respect to the BCC O-ones.

In general, in a given circumstance, hydrogen prefers one type of interstitial site with respect to all the other available ones. In case of palladium, the preferred site are the octahedral ones. Understanding why a certain type of site is preferred over another one would allow getting crucial information on the diffusion mechanism of hydrogen into metals [13].

The interstitial sites represent a finite population, whose occupancy by the dissolved hydrogen can be described by means of a thermodynamic approach in terms of Fermi-Dirac statistics. Smirnov and Pronchenko provided a detailed expression of chemical potential of the Pd-H system, taking into account three different contributions: (i) conformational, (ii) oscillatory and (iii) electronic. Then, chemical potential can be obtained as the derivative of the Helmholtz free energy (*F*) with respect to the hydrogen molar fraction *ξ* in the metal lattice (Equation (1)), which is defined as the hydrogen/interstices concentration ratio [22].

(1) $$\begin{array}{l} \mu =\frac{\partial F}{\partial \xi }=\mu_{conf}+\mu_{osc}+\mu_{el}\\ =\frac{\partial F_{conf}}{\partial \xi }+\frac{\partial F_{osc}}{\partial \xi }+\frac{\partial F_{el}}{\partial \xi } \end{array}$$

The conformational chemical potential can be written as described in Equation (2):

(2) $$\mu_{conf}=RT\ln\frac{\xi }{1-\xi }+U_{H}^{conf}+U_{\mathrm{HH}}^{conf}\xi$$

where $U_{H}^{conf}$ and $U_{\mathrm{HH}}^{conf}$ are the bond energy of atom H with the metal lattice and the H-H interaction energy within the interstices. The oscillatory contribution to the Helmholtz free energy is reported in Equation (3) [23]:

(3) $$F_{osc}=3RT\xi \ln\left(2\sinh\frac{\hbar \omega \left(\xi \right)}{2RT}\right)\;$$

from which the corresponding expression of chemical potential can be obtained as follows (Equation (4)):

(4) $$\mu_{osc}=\frac{\partial F_{osc}}{\partial \xi }=3RT\ln\left(2\sinh\frac{\hbar \omega \left(\xi \right)}{2RT}\right)-3b_{1}\xi \coth\frac{\hbar \omega \left(\xi \right)}{2RT}\;$$

The parameter ω is the frequency of local oscillations of H-atoms, which depends on H-concentration. Smirnov et al. suggested the following linear approximation for ω (Equation (5)) [22]:

(5) $$\hbar \omega \left(\xi \right)=\hbar \omega_{0}-b_{1}\xi \Rightarrow \underset{\xi \to 0}{\lim}\hbar \omega \left(\xi \right)=\hbar \omega_{0}\;$$

As for the electronic contribution to chemical potential, Smirnov and Pronchenko proposed the following two-parameter expression valid in the entire H-content range within an approximation of 5% (Equation (6)) [22]:

(6) $$\mu_{el}=A\xi +B\xi^{4},\;0\leq \xi \leq 1\;$$

From the previous expressions, the following complete expression of chemical potential is obtained (Equation (7)):

(7) $$\begin{array}{ll} \mu = & RT\ln\frac{\xi }{1-\xi }+U_{H}^{conf}+U_{\mathrm{HH}}^{conf}\xi +3RT\ln\left(2\sinh\frac{\hbar \omega \left(\xi \right)}{2RT}\right)\\ & -3b_{1}\xi \coth\frac{\hbar \omega \left(\xi \right)}{2RT}+A\xi +B\xi^{4} \end{array}$$

Once chosen an appropriate reference chemical potential *μ*_0_, the corresponding activity of atomic hydrogen in the metal lattice is defined (Equation (8)):

(8) $$\mu =\mu_{0}+RT\ln a\left(\xi \right)$$

In the present paper, to obtain a complete expression of hydrogen activity in the metal bulk, the chemical potential in the infinite dilution conditions (i.e., hydrogen content tending to zero) is chosen as the reference chemical potential (Equation (9)):

(9) $$\mu_{0}=\mu \left(\xi \to 0\right)=3RT\ln\left(2\sinh\frac{\hbar \omega_{0}}{2RT}\right)$$

From Equations (8) and (9), an explicit expression of the H-activity is finally obtained (Equation (10)):

(10) $$\begin{array}{ll} a\left(\xi \right)=\exp\left(\frac{\mu -\mu_{0}}{RT}\right)= & \frac{\xi }{1-\xi }\exp\left(\frac{U_{H}^{conf}}{RT}+\frac{U_{\mathrm{HH}}^{conf}}{RT}\xi \right)\times {\left(-2\sinh\frac{b_{1}\xi }{2RT}\right)}^{3}\\ & \times \exp\left(-\frac{3b_{1}}{RT}\xi \coth\frac{\hbar \omega \left(\xi \right)}{2RT}+\frac{A}{RT}\xi +\frac{B}{RT}\xi^{4}\right) \end{array}$$

In the limit of infinite dilution system (*ξ* → 0), the activity *a* coincides with the hydrogen composition *ξ*. Based on Equation (7), as *ξ* tends to the limit value of 1, the chemical potential *μ* tends to infinity. From a physical point of view, as the available sites are gradually more occupied, it becomes progressively more difficult to fill the remaining sites, which corresponds to a situation of a gradually higher chemical potential.

From the energetic point of view, the hydrogen atoms progressively occupy the interstitial sites from the lowest-energy class to the highest-energy one [20].

Indeed, despite a former theory, which expected the occupancy of just one class of sites, i.e., the octahedral ones in a Pd-H system, McLennan et al. confirmed through diffraction evidence that, for the palladium-deuterium system, the tetrahedral sites, which are the highest-energy class of sites, are partially occupied when the deuterides PdD_x_ are formed above the thermodynamic critical point. Moreover, the same authors found via Rietveld profile analysis that the maximum tetrahedral occupancy occurs at a D/Pd atomic ratio of around 0.6, at which one third of all D-atoms are placed in tetrahedral sites [24].

An explanation for these observations can be provided if considering that the interactions between the H-atoms dissolved in the metal bulk induce an energetic difference between occupied and empty sites belonging to the same class. In particular, it is proposed that the hydrogen atoms occupies tetrahedral sites on alternate planes, forming in this way a symmetric, ordered and more energetically favorable sub-lattice, as occurs in tantalum–hydrogen system [20].

The occupation of the interstitial sites induces a stress state [20,25,26]. In fact, the metal lattice expands upon H_2_ absorption. To intuitively understand how the occupation of the interstices occurs, it is useful to consider the *ball-in-hole* model in continuum elasticity. In this model, the hydrogen atom is assumed to be an incompressible ball, while the metal cavity in which the ball fits is thought as an elastic, spherical *hole*. When the ball enters the hole, the hole size increases because of the ball-hole interactions, inducing an additional volume metal matrix. If Δ*V*_1_ is the volume of the intruding ball, the Δ*V*_2_ volume of the cavity is given by:

(11) $$\Delta V_{2}=\frac{3\left(1-\nu \right)}{1+\nu }\Delta V_{1}$$

where *ν* is the Poisson ratio of the matrix. For a typical value of *n*_H_/*n*_Pd_ of 0.3, it results that Δ*V*_2_ is greater than Δ*V*_1_ by 50%. Thus, the volume of the body increases by an extent larger than that required to accommodate an H-atom: this is the origin of the increase of the lattice parameters caused by the insertion of dissolved H-atoms [20].

**Figure 2:**
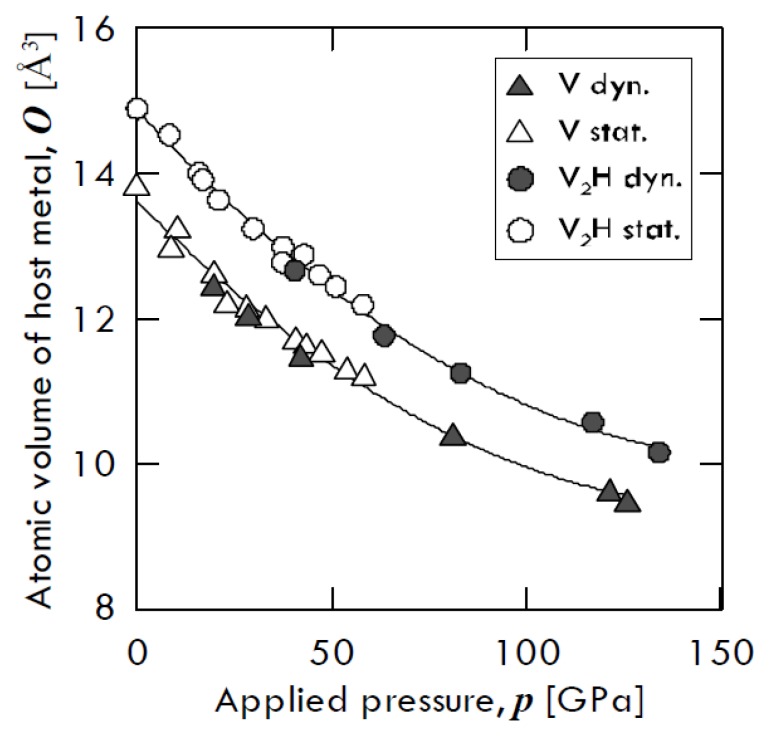
Compression curves of *β*-V_2_H and pure vanadium. The open symbols refer to static compression data (diamond anvil cell), while the closed ones refer to dynamic compression data (shock wave). Adapted from Fukai [13].

Besides, Fukai [13] found that hydrogen-induced augmented volume is basically incompressible. The compression curves of pure vanadium and that of *β*-V_2_H hydride, obtained by means of two different compression ways—i.e., through diamond anvil cell (open symbols) and through shock wave (closed symbols)—are shown in Figure 2. From this figure, one can notice that both vanadium and V_2_H trend keep almost the same atomic volume gap. In particular, pure V and V_2_H volume decrease of about 69% and 74% of its original value at 130 GPa, respectively, with the two values being relatively close to each other.

It must be remarked that, although the same amount of hydrogen could be included into the metal lattice, the method used to inject hydrogen could affect the lattice distortion and, thus, the internal thermodynamic state of the metal-solute system. Indeed, inserting hydrogen simply by applying a pressure difference contrasts the lattice expansion, whereas the expansion effect is not hindered when metal is subject, for example, to cathodic charging [20].

### 2.2. Elastic and Electronic Effects of Alloying

The lattice constants are different for different systems as well as the volumes of the tetrahedrons. The larger the volume, the wider the free space available for the dissolution of the interstitial atoms, which thus results facilitated.

When an alloying element is inserted in the metal lattice, the lattice constants could change, affecting the solubility of hydrogen. Lu et al. [21] simulated the behavior of hydrogen in a V-based 2 × 2 × 2 supercell made by 8 unit cells, in which an alloying M-atom (Al, Ti, Cr, Fe, Ni and Nb) replaces the central V-atom of such a supercell, thus forming a V-based alloy. This alloy is indicated as V_15_M, because 15 V-atoms and a single M-atom form the unit cell lattice.

When an alloying element is added to vanadium, its atomic radius does not match the vanadium original matrix, leading to lattice distortions and change in the volume of tetrahedrons. These distortions produce an elastic stress field, which affects the H-atoms dissolution, resulting in the variation of the solution energies. Such variations were found to depend significantly on the lattice constants of V_15_M, which in their turn depend on the size of alloyed atoms [21].

**Figure 3:**
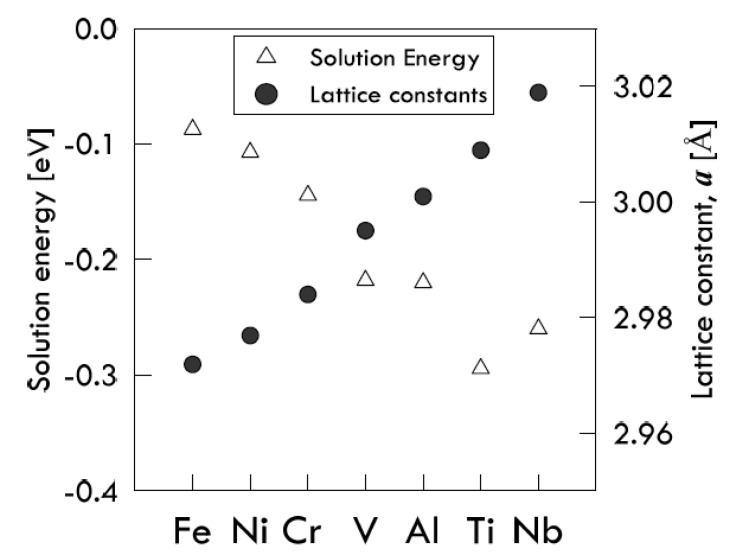
Solution energy of hydrogen in V_15_M alloys, compared with the lattice constants of each alloy. Adapted from Lu et al. [21].

As the M-atom size increases, the cubic lattice tends to swell to a certain extent, thus causing the available space for the dissolution of an additional H-atom to increase as well. In this way, the M-atoms having a size smaller than the V-atom one (like Cr, Ni and Fe) reduce the solubility of hydrogen in vanadium by making the solution energy values less negative. The opposite occurrence is found for M-atoms with a bigger size (Al, Ti, Nb) [21]. Results are illustrated in Figure 3.

A remarkable exception to the previously explained general cases is found by observing the behavior of niobium. In fact, since it is the biggest atom in the analyzed series, it is expected to form an alloy with a solution energy more negative than that of titanium, which has a smaller atomic size. However, such an expectation is not confirmed by simulation. Those authors explain this apparently anomalous behavior by considering that the effective available space in the interstices of the V-based lattice is reduced by the size of the Nb-atom, which is too large compared with the V-atom one [21]. The addition of an alloying atom into the vanadium lattice affects the interstitial H-diffusion not only by producing elastic distortion, but also by modifying the original electronic structure around the interstitial solute. Investigating the local density of states, however, Lu and his co-workers found that the intensity values of the resonance peaks of the hydrogen in the pure vanadium system are similar to those of the hydrogen in V_15_M system. From this observation, it is withdrawn that the electronic effect due to the M-atom presence may be not the main influencing factor of hydrogen dissolution [21].

### 2.3. Effects of Dissolved Atoms on Global Electronic Structure

The enthalpy of hydrogen dissolution $\Delta {\bar{H}}^{0}$ from the molecular state to the atomic one inside metal is given by the contribution of two terms. The first is related to the dissociation energy of the molecule and the second is related to the interaction energy between dissolved atoms as well as between atoms and metal. In particular, the metal–atoms interaction determines the metals classification into two classes of occluders: endothermic and exothermic ones. The former has a solubility increasing with increasing temperature, whereas the latter shows the opposite behavior [20].

Endothermic occluders, such as Mn, Fe, Co and Ni, form a metal–hydrogen solid solution in which the H-atom is located at random interstices [11]. However, the most interesting transition metals for hydrogen separation, e.g., Pd, Ta, V, Nb, are exothermic occluders, which show a tendency to form ordered hydrides at high H-concentrations [27].

**Figure 4:**
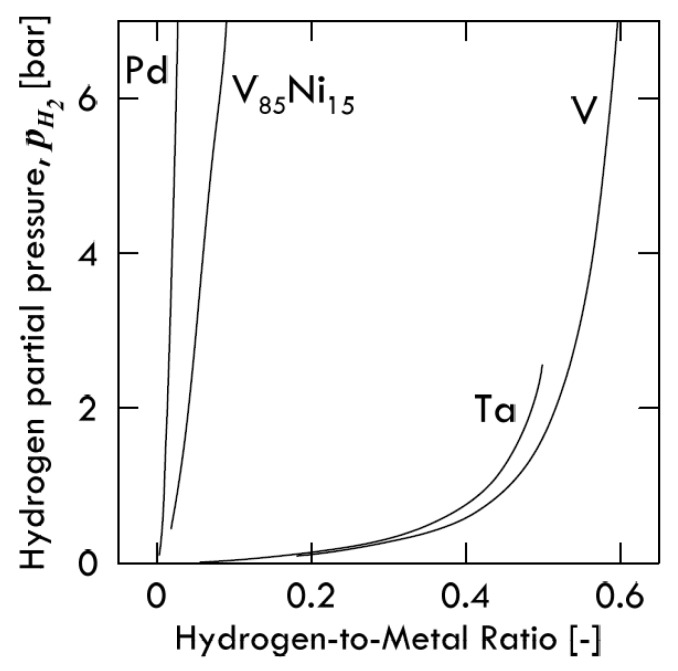
Isotherms for selected metals and alloys measured using Sieverts’ technique, in which the metal sample is equilibrated in a hydrogen atmosphere. The test for Pd are carried out at 340 °C, whereas for all the others metals a temperature of 400 °C is used. Adapted from Dolan [11].

The solubility isotherms of some metals and a V-alloy (vanadium, tantalum and V_85_Ni_15_ alloy at 400 °C, and palladium at 340 °C) are shown in Figure 4. As is possible to notice, vanadium clearly absorbs much more hydrogen than palladium. Under a hydrogen partial pressure of 7 bar, the H/V ratio at 400 °C would be around 0.6, i.e., 6 hydrogen atoms per 10 vanadium atoms, while, at the same pressure, the H/Pd ratio at 340 °C would settle at about 0.02 (ca. 2 ÷ 98). Since exothermic occluders show a solubility decreasing with increasing temperature, the difference between these two metals would become even more pronounced with palladium at 400 °C. Therefore, it is possible to state that the dominant contributor to permeability is solubility for BCC metals, whereas for FCC metals (Pd in particular), the dominant factor is represented by diffusivity [11].

Consequently, permeability of BCC metals decreases with increasing temperature, because this behavior is driven by solubility. On the contrary, permeability of FCC metals increases with increasing temperature, because it mainly driven by diffusivity. It is interesting to underline how alloying vanadium with a small percentage of nickel drastically reduces hydrogen solubility. At the same condition of pressure and temperature, the hydrogen/metal ratio would pass from 0.6 to 0.09. By reducing the hydrogen solubility, the embrittlement tendency reduces as well [11].

The H-content in the metal lattice affects also other physical properties, such as electrical resistance, magnetic susceptibility and thermoelectric power. For their extent, these variations cannot be simply explained by lattice expansion induced by solute atoms. Differently, it is clear that hydrogen consistently perturbs the electronic structure of the host metal [20]. Indeed, the *1s* electrons of the hydrogen atoms enters the *s-* and *d-* bands of the host metal, causing shifts in the energy bands. As a result, the Fermi electrons of the host metal start surrounding the positive H^+^ nuclei, leading to the formation of a sort of *screened entity*, which could be thought as neutral atoms even if the electrons are not bound to the nuclei. Accordingly, it is possible to distinguish two kinds of effects. The first consists in the shift of metal bands, which has a *long-range* effect, as the induced interactions involve the entire metal domain. The second is related to the heaping of Fermi electrons placed on the hydrogen nuclei and is a *short-range* effect. An attractive interaction is observed between two hydrogens caused by the *d-*vacancies of the Pd-atom, which is the common neighbor of both H-atoms. This interaction could explain the elastic interactions as well as the hydrogen cluster formation in Pd-H systems. Since hydrogen remarkably affects the global electronic structure and, more importantly, the metal atoms inter-distance, it is not surprising that the cohesive forces of the host metal are unavoidably reduced. Such an occurrence is explained through molecular dynamic simulations, which show that hydrogen fills antibonding states in *4d* band, reducing the bond strength between Pd-atoms. The decrease of the electron density between metal atoms, resulting in the Pd-Pd bonds impairment in several transition metals, is confirmed through cluster calculation and embedded atom method [20].

### 2.4. Hydrogen-Lattice Defects Interactions

Hydrogen can also interact with structural defects. Metals usually exhibit defects of various size, which can be distinguished into three classes, so-called *zero-dimensional*, *one-dimensional*, and *two-dimensional* one.

**Figure 5:**
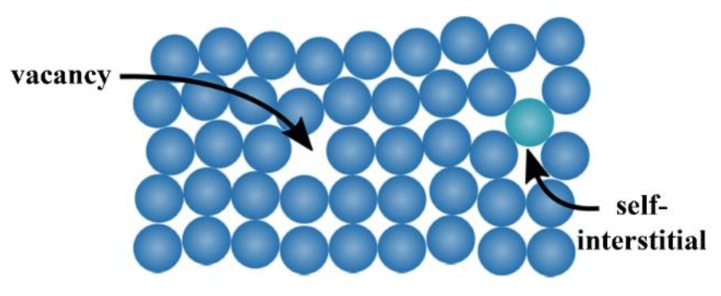
Point defects in a crystal structure: vacancy and self-interstitial/substitutional atom.

The zero-dimensional defects, also known as *point defects*, are local defects, i.e., locations where an atom is missing (vacancy) or is placed into an interstitial void of the lattice structure (self-interstitial), as illustrated in Figure 5. The latter occurs only at low concentration because of the strain field induced in the tightly packed metal structure, whereas the former can be found more often, with a probability increasing with increasing temperature. In fact, the higher the temperature, the more frequent and random the position changes of the atom in the lattice. It can also happen that the vacancies population is in excess with respect to the equilibrium value, for example after a quenching from high temperature. Anyway, both defect types can also occur after plastic deformations.

**Figure 6:**
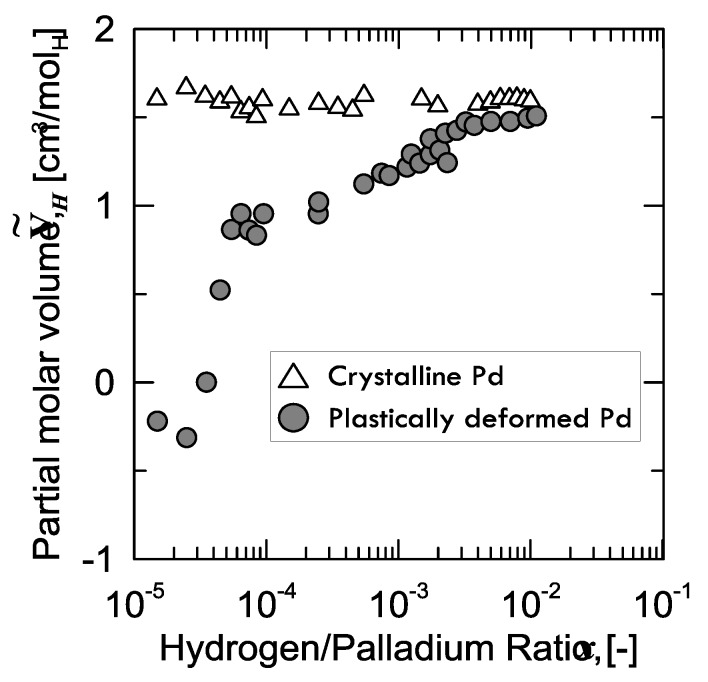
Partial molar volume of hydrogen measured in crystalline Pd (open triangles) and in plastically deformed Pd (closed circles) by cold drawing and rolling, as a function of hydrogen concentration in palladium (number of H-atoms per number of Pd-atoms). Adapted from Kirchheim [28].

Regarding the interaction between these defects and H-atoms, a curious aspect was observed by Kirchheim [28], who prepared two samples of palladium: the first one, which was structurally defect-free, and the second one plastically stretched (up to 50%) through cold drawing and subsequent rolling, in order to enhance the number of defects. Afterwards, Kirchheim measured the partial molar volume ${\tilde{V}}_{H}$ of hydrogen in both samples (Figure 6).

It can be observed that, for the virtually defect-free Pd-sample, ${\tilde{V}}_{H}$ is constant within the whole range of H/Pd ratio, whereas the plastically deformed one shows a continuously increasing value tending to the same level as that of the former.

A peculiar aspect to be observed is that, for the defected sample, ${\tilde{V}}_{H}$ is even negative below a certain value of hydrogen concentration (40 ppm). In other words, instead of expanding because of the hydrogen absorption, the lattice shrinks. Kirchheim explained this weird behavior considering the phenomena occurring when hydrogen fills an empty site or vacancy. In fact, if hydrogen is close enough to the nearest palladium atom at low concentration, attractive forces Pd-H come into play and, thus, the lattice contracts. From around 40 ppm on, the lattice starts growing in the positive values, as the effect of hydrogen in the substitutional vacancies is overcome by the expansion of the normal interstitial sites [28].

The one*-*dimensional defects, also known as linear defects, consist in lattice local dislocations, produced in the metal by plastic deformations and many other processes. One can basically distinguish two types of dislocations: the *edge* dislocation and the *screw* dislocation, even if most dislocations are probably a hybrid of these two limits.

**Figure 7:**
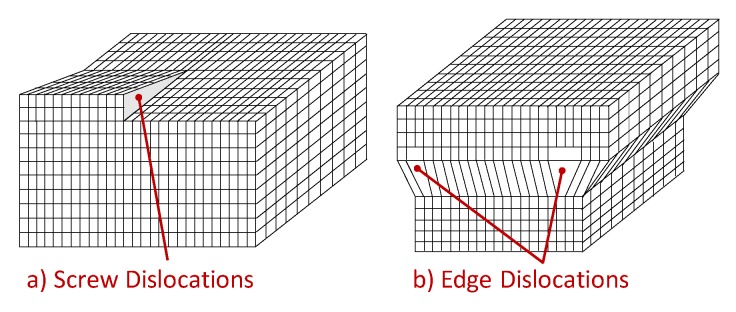
Illustration of linear defects: (**a**) Screw dislocation and (**b**) Edge dislocation.

The shear stress causes screw defects, which propagates along a direction normal to the shear stress, as depicted in Figure 7a. As well, the edge dislocations can be visualized as an extra half-planes inserted in the crystal homogeneous lattice, as sketched in Figure 7b. The interatomic bonds are significantly distorted but only in the immediate neighborhood. When a shear stress is applied, dislocations can not only originate but also move. It is important to go deeper into this phenomenon, to understand the hydrogen dragging induced by dislocation motion. To make an analogy, this type of movement can be compared to the motion of a caterpillar: first, it moves the rear part creating a hump, and then moves the front part, similar to how the defect slips one cell per time. In this way, only a small amount of bonds is broken at a given time, thus requiring a much smaller energy than that needed to break all the bonds simultaneously. For the same reason, dislocations move easily over dense planes, because the stress required to move the dislocations increases with increasing spacing between planes. As FCC and BCC metals have several dense planes, the dislocations move relatively easily, resulting in materials with a relatively high ductility.

Kirchheim [29] evaluated the interaction energy of hydrogen in palladium edge dislocations. He found that the closer the core of the edge defect, the larger the interaction energy, which includes a self H-H attractive interaction energy. Far from the cores, the H-H interactions do not provide an appreciable contribution and, in general, the attractive interaction energy decreases with the reciprocal of the distance. Anyway, the additional population of hydrogen can be measured only at very small quantities of dissolved hydrogen due to the attractive interaction with edge dislocations. When carbon embeds in *α*-Fe, it leads to tetrahedral distortions causing attractive interactions towards screw dislocations. On the contrary, the octahedral positions occupied by the H-atoms in palladium make it interact with this type of dislocation at negligible or null levels [29].

The two-dimensional or planar defects include several forms of dislocations. Overall, stacking faults, grain boundaries and internal surfaces of micro-voids and micro-cracks are mentioned. To explain the stacking fault phenomenon, one can consider the case of the crystalline structure of two common lattices, the FCC and the HCP structures, for which the former can be oriented in two different planes, i.e., square or triangular, whereas the latter can be seen in the triangular orientation. In particular, one can imagine the crystal structure as a sequence of stacks. If A indicates the triangle, B the hexagon and C the reverse triangle, one can describe the HCP structure as an AB sequence, whereas the FCC structure as an ABC sequence. With this in mind, a stacking fault occurs, for example, when an unpredicted C-layer is present in the AB sequence of the HCP structure or when a C-layer is missing, or ABC default sequence randomly changes in the FCC structure.

In their study, Whiteman and Troiano showed a hydrogen-charging operation in a stainless-steel sample (25%Cr–20%Ni) conducted through electrolytic way. In those conditions, they found that the activation energy required to create a stacking fault lowers. Such an energy variation can be associated with an attractive interaction between hydrogen dissolved in metal and stacking faults [30]. Another type of planar defect is represented by the grain boundary.

**Figure 8:**
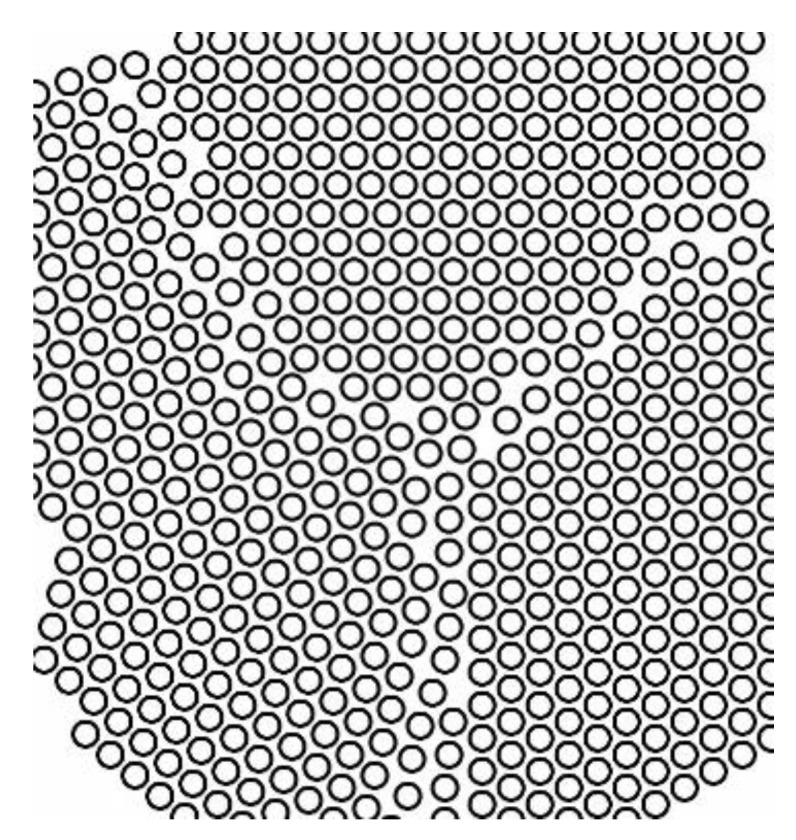
Illustration of grains and relative boundaries in a crystal.

Generally, a solid consists of several crystallites or grains, whose size varies from nanometers to millimeters. Each grain has usually a different orientation with respect to neighboring grains. The border separating one grain from another one is known as grain boundary. A representation of the grain boundaries present in crystal is sketched in Figure 8.

**Figure 9:**
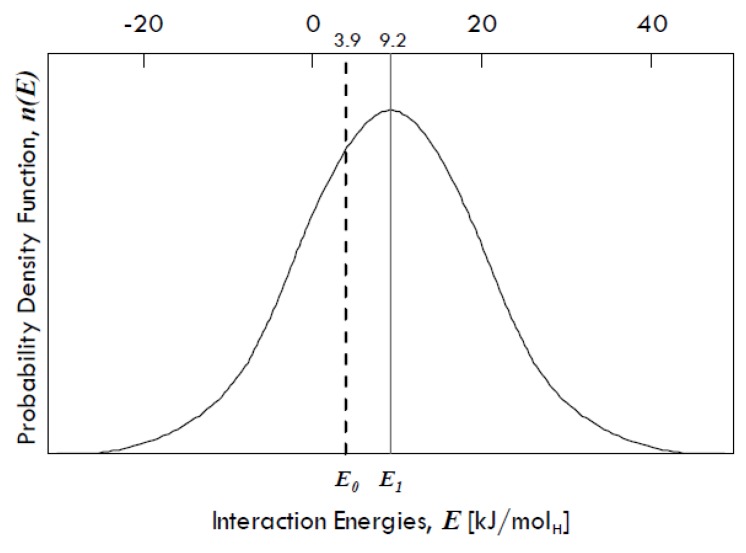
Gaussian distribution of site energies for hydrogen in nano-crystalline palladium. Adapted from Mütschele and Kirchheim [31].

Grain boundaries have been demonstrated to absorb an excess of H-atoms population in several metals other than palladium. Mütschele and Kirchheim [31] found that there is a Gaussian distribution of interaction energies between dissolved hydrogen and grain boundaries in palladium (Figure 9), denoting a variety of binding sites in grain boundaries.

The energy *E*_1_ is equal to 9.2 kJ/mol_H_ and represents the expected value (*µ*) of the interaction energies distribution around which site energies for the grain boundaries might vary, whereas *E*_0_ is equal to 3.9 kJ/mol_H_ and represents the interaction energy of the sites within the grains, which has the same value as that of the single crystals of Pd. The difference between these two energies (equal to 5.3 kJ/mol_H_) gives the spectrum of the segregation energies, i.e., the energies of molecular partitioning from metal bulk to defects.

The standard deviation of the distribution (*σ*) is equal to 15 kJ/mol, so that it is possible that some grain boundary sites show even a negative interaction energy, which means that there can be also repulsive forces. However, grain boundaries in any metal exhibit in general attractive interactions towards dissolved hydrogen [31].

In addition to stacking faults and grain boundaries, a third class of defects is represented by micro-voids, which can occur when a ductile material is pulled during a tensile strain. If the stress continues, a coalescence of some voids can occur, leading to the formation of micro-cracks. Hydrogen is absorbed upon these cracks in the same way as if it would be absorbed upon a generic external surface. In addition, the volume of the internal cavity (which can be referred to as a *three-dimensional* defect) would acquire a concentration of molecular hydrogen consistent with the input pressure of external environment at equilibrium [20]. It is possible to divide the interactions between dissolved hydrogen and lattice imperfections into two categories: chemical and elastic one. The former refers to positions where the atomic displacements are remarkable, e.g., at dislocation cores and internal interfaces, whereas the latter refers to positions where atomic displacements are small, which means sufficiently far from the imperfections. In this case, an approximated linear elasticity model can be applied [20].

## 3. Phase Diagrams and Phase Transitions

### 3.1. Thermodynamics of Hydrogen Dissolution in Metal Membranes

When the hydrogen mass transport is limited by the bulk diffusion (i.e., the diffusion in the metal lattice) as occurs in most cases, permeability is the best way to compare the intrinsic transport properties of different materials. This parameter can be seen as the overall result of the effect of two intrinsic properties: diffusivity and solubility, both crucially depending on the gas/solid system.

**Figure 10:**
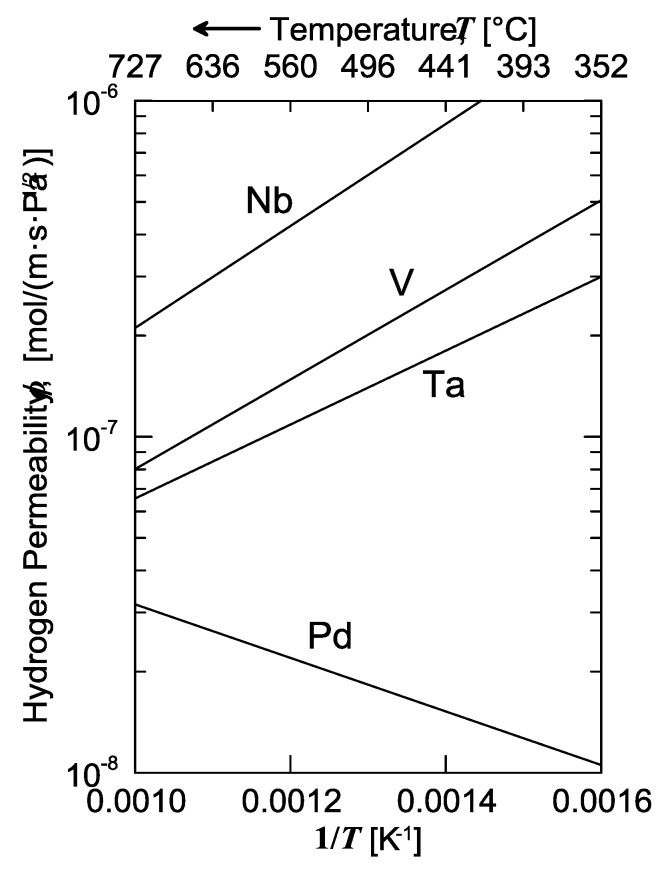
Permeability of selected metals as a function of temperature. Adapted from Buxbaum and Kinney [32].

The temperature dependence of permeability of several transition metals is shown in Figure 10. We can observe that those metals with the highest permeability are early transition metals (like niobium, vanadium and tantalum) with a BCC structure. In terms of the sole permeability, those materials could easily overcome the performances of palladium. However, in practice, the major barrier preventing from achieving such a high flux is the embrittlement by hydrogen [11], which is the macroscopic result of all the above-discussed phenomena generating micro-cracks, dislocations and local strains eventually leading to mechanical failure of the metal lattice. Furthermore, an important phenomenon to understand is the permeability change with temperature. While permeability increases with increasing temperature for palladium, the BCC metals show the opposite behavior. This effect can be explained by considering the solubility influence. As solubility represents the strength of the H-metal interactions usually evaluated by measuring the amount of dissolved species, it generally decreases with increasing temperature, mainly because of the stronger lattice vibrations, which favor the release of the solute from the interstices.

In the hypotheses of the validity of the Langmuir adsorption model—i.e., mono-layer adsorption, energetically uniform adsorption sites, no lateral interactions among adsorbed atoms and ability of each adsorption sites to accommodate a single molecule/complex [33]—and in the absence of any inhibiting and/or poisoning species, the hydrogen surface coverage on feed and permeate side can be respectively expressed by Equation (12) [34]:

(12) $$\theta_{H}^{}={\frac{K_{H_{2}}^{}\sqrt{P_{H_{2}}^{}}}{1+K_{H_{2}}^{}\sqrt{P_{H_{2}}^{}}}|}_{\begin{array}{l} \mathrm{Feed}\\ \mathrm{Perm} \end{array}}\;$$

From the equilibrium condition between surface and bulk Pd-H systems, which is always assured in the operating conditions of interests for hydrogen purification applications [35,36], we have Equation (13) [37]:

(13) $$\xi ={\frac{a_{1}\left(T\right)\theta_{H}^{}}{a_{1}\left(T\right)\theta_{H}^{}+\left(1-\theta_{H}^{}\right)}|}_{\begin{array}{l} \mathrm{Feed},\\ \mathrm{Perm} \end{array}}\;$$

Combining Equations (12) and (13), an explicit relationship expressing the H-concentration in the metal bulk as a function of the hydrogen partial pressure in the gas phase is obtained (Equation (14)).

(14) $$\xi ={\frac{a_{1}{\left(T\right)}^{}\frac{K_{H_{2}}^{}\sqrt{P_{H_{2}}^{}}}{1+K_{H_{2}}^{}\sqrt{P_{H_{2}}^{}}}}{a_{1}\left(T\right)\frac{K_{H_{2}}^{}\sqrt{P_{H_{2}}^{}}}{1+K_{H_{2}}^{}\sqrt{P_{H_{2}}^{}}}+\left(1-\frac{K_{H_{2}}^{}\sqrt{P_{H_{2}}^{}}}{1+K_{H_{2}}^{}\sqrt{P_{H_{2}}^{}}}\right)}|}_{\begin{array}{l} \mathrm{Feed},\\ \mathrm{Perm} \end{array}}\;$$

In the limit of Sieverts’ hypotheses, the following approximation can be made (Equation (15)):

(15) $$\xi ≅{a_{1}\left(T\right)K_{H_{2}}^{}\sqrt{P_{H_{2}}^{}}|}_{\begin{array}{l} \mathrm{Feed},\\ \mathrm{Perm} \end{array}}\;$$

which leads to the well-known Sieverts’ law if the constant $K_{H_{2}}$, which depends on temperature as well, is the same for both membrane side. This fact underlines that the use of an empirical Sieverts’ law to get information on the non-ideal behavior of the hydrogen permeation through metal membranes should be used carefully to correctly interpret the experimental results at relatively high pressure and in the presence of other external phenomena [38,39].

Substituting Equation (14) into Equation (10), we have an explicit expression of the activity of the H-atoms dissolved in the lattice (H(*sol*)) as a function of the hydrogen partial pressure (Equation (16)):

(16) $$a\left(\xi \right)\equiv a\left[H\left(sol\right)\right]\Rightarrow a\left(P_{H_{2}}^{}\right)\;$$

The expression of the H-activity is needed to solve the equilibrium of the solution process of hydrogen in the metal lattice (*r_ov_*), which is actually the overall result of two different processes, i.e., the dissociative adsorption of hydrogen onto the surface (*r*_1_) and the solubilization (or dissolution) of the adsorbed H-atoms into the metal bulk (*r*_2_).

(17) $$\begin{array}{l} r_{1}:\\ r_{2}:\\ r_{ov}: \end{array}\begin{array}{l} H_{2}\left(g\right)=2H\left(ads\right)\\ \underline{H\left(ads\right)=H\left(sol\right)}\\ H_{2}\left(g\right)=2H\left(sol\right) \end{array}\;$$

The equilibrium constant of the overall solubilization process can be expressed in terms of activities as follows (Equation (18)):

(18) $$\begin{array}{l} K_{e,ov}=\exp\left(-\frac{\Delta {\bar{G}}_{r_{ov}}^{0}\left(T\right)}{RT}\right)=\frac{a{\left[H\left(sol\right)\right]}^{2}}{a\left[H_{2}\left(g\right)\right]}\\ \Delta {\bar{G}}_{r_{ov}}^{0}\left(T\right)=\Delta {\bar{H}}_{r_{ov}}^{0}\left(T\right)-T\Delta {\bar{S}}_{r_{ov}}^{0}\left(T\right) \end{array}$$

where the Gibbs free energy of the overall reaction as well as its enthalpy and entropy can be actually written as the appropriate linear combinations of the corresponding thermodynamic state functions of the reactions *r*_1_ and *r*_2_ (Equation (19)):

(19) $$\begin{array}{l} \Delta {\bar{G}}_{r_{ov}}^{0}\left(T\right)=\Delta {\bar{G}}_{r_{1}}^{0}\left(T\right)+2\Delta {\bar{G}}_{r_{2}}^{0}\left(T\right)\\ \Delta {\bar{H}}_{r_{ov}}^{0}\left(T\right)=\Delta {\bar{H}}_{r_{1}}^{0}\left(T\right)+2\Delta {\bar{H}}_{r_{2}}^{0}\left(T\right)\\ \Delta {\bar{S}}_{r_{ov}}^{0}\left(T\right)=\Delta {\bar{S}}_{r_{1}}^{0}\left(T\right)+2\Delta {\bar{S}}_{r_{2}}^{0}\left(T\right) \end{array}$$

In Equation (18), the activity of the dissolved hydrogen can be evaluated using Equation (16), whereas that of the gaseous hydrogen can be made explicit by using fugacity, which can be expressed in terms of hydrogen partial pressure and fugacity coefficient evaluated by an appropriate equation of state (Equation (20))

(20) $$K_{e,ov}=\frac{a{\left[H\left(sol\right)\right]}^{2}}{\frac{f\left[H_{2}\left(g\right)\right]}{f^{0}=1\;\mathrm{bar}}}=\frac{a{\left[H\left(sol\right)\right]}^{2}}{\frac{\phi \left[H_{2}\left(g\right)\right]P_{H_{2}}}{f^{0}=1\;\mathrm{bar}}}\;$$

In this way, in pure-hydrogen conditions, the right-hand side of Equation (20) can be expressed as a function of the hydrogen partial pressure only, whose complexity requires the implementation of an appropriate numerical method to obtain a solution.

### 3.2. PCT Diagrams

Since one of the major characteristics of the exothermic occluders is the formation of a hydride, it is possible to represent the equilibrium conditions of this type of metal–hydrogen system by means of a phase diagram, analogously to what done for the phase diagrams used to represent the metal alloy systems [27]. In particular, the formation of metal hydrides leads to the existence of different phases in the palladium–hydrogen system: *α*-phase and *β*-phase.

The *α*-phase is a solution phase and has lattice constants close to those of palladium. At room temperature, the H: Pd ratio for this phase is 0.03. As more hydrogen dissolves in metal, the lattice constants are observed to increase linearly with pressure until the *β*-phase (metal hydride) appears. However, the situation is actually more complex, since the transition from *α*- to *β*-phase is not sharp but passes through the existence of intermediate phases composed of a mix of both. Moreover, hysteresis is observed in increasing/decreasing cycles of hydrogen concentration [40]. The composition of the *β*-phase is approximately 0.6 at room temperature [41], which is considerably higher than the value of the *α*-phase. Both *α*- and *β*-phase show the same FCC lattice structure, with the same octahedrally coordinated H-atoms [42]. Please note that the same sites are occupied by hydrogen in *α*-palladium hydride at a considerably lower H: Pd ratio.

For the *α*-phase at room temperature (20 °C), the value of the H:Pd ratio is 0.008, while for the *β*-phase such a value is 0.607. For this reason, even if the *α*- and *β*-phase have the same lattice symmetry, the specific volumes are different: at room temperature, the values of the lattice constant *a* referred to the side of cubic unit cells for both phases are reported in Table 1. As is possible to notice, volume increases up to 10% in the *α*-to-*β* transition, determining a change of molar volume equal to 1.57 cm^3^/mol_H_ [43]. Such a transition involves the formation of clusters of H-atoms such that 125 Pd-atoms have nearly 76 H-atoms in the *β*-phase and just one H-atom in the *α*-phase [20]. Therefore, for the performance of membrane-assisted purification processes, monitoring of the formation of the *β*-phase is crucial, as it leads to a larger volume expansion as well as higher internal stresses that need be relaxed to avoid cracks and fractures [11].

**Figure 11:**
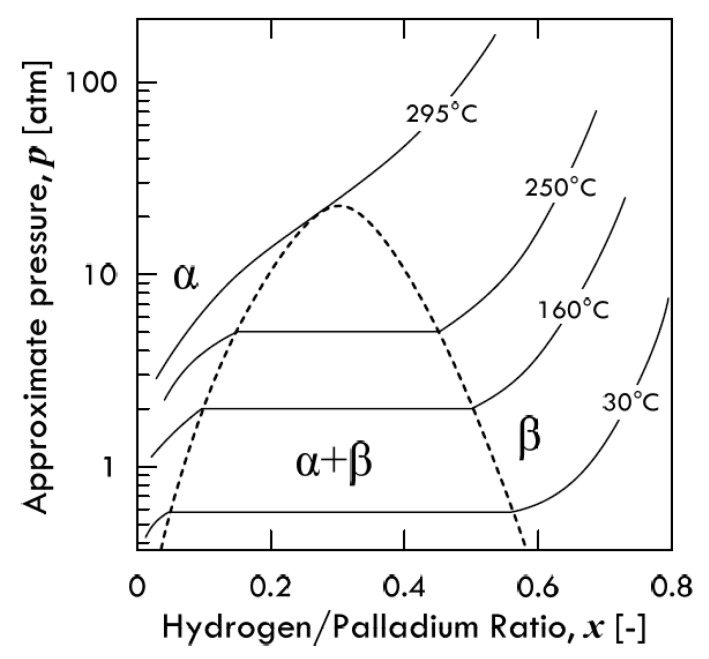
Approximate Pressure-Composition-Temperature phase diagram for the Pd-H system. Adapted from Knapton [45].

In general, metal–hydrogen systems exhibit phase diagrams of various complexities. Phase transitions have been object of study since the first part of the 20th century, when Gillespie and Galstaun [44] first described the *Pressure-Composition-Temperature* (PCT) diagram for palladium (Figure 11). Specifically, they found a critical point at 295.3 °C and 19.8 atm, above which only a single phase exists. Therefore, such a critical point represents the highest temperature at which the *β*-phase can form. In the single-phase region, the composition varies smoothly, and the distortion phenomena are circumvented. Therefore, one method whereby the phase change can be avoided is to ensure that the palladium membrane is always operating within the single-phase region of the Pressure-Composition isothermal diagram [45].

**Figure 12:**
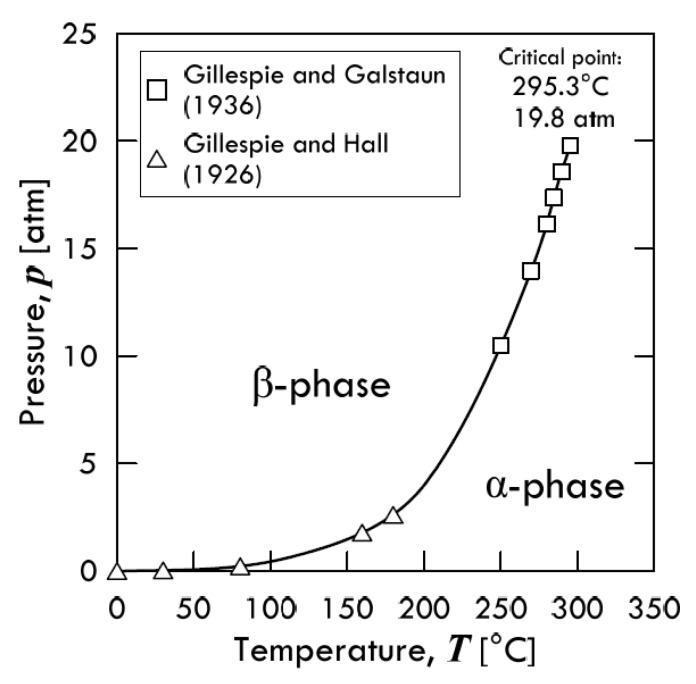
Pressure-Temperature phase diagram for the Pd-H system. Adapted from Jewell et al. [41].

The bell-shape curve shown in Figure 11 takes the name of *coexistence curve*, since it borders a miscibility gap where the *α*- and *β*-phase can coexist. Such a PCT diagram for the PdH_x_ system could be simplified in a PT diagram, as shown in Figure 12. In this graph, the mixed (*α* + *β*) area is not shown, and only the boundary between the two single phases is visible. Similar to palladium, also in the PCT diagrams of vanadium [46], niobium [47] and tantalum [48] isotherms exhibit the plateau typical of exothermic occluders. This plateau is due to the re-ordering of *α*-to-*β* transition in octahedral interstices [11]. The critical temperatures for each metal are reported in Table 2 along with the related H:M ratio.

### 3.3. Coherent and Incoherent States

The miscibility boundaries of the coexistence curve are influenced by the concentration of hydrogen in the metal. This effect is related to differences in zero-point energies, in lattice expansion and in phonon spectrum. Besides, a modification of the coexistence curve can be also related to the effects of lattice stresses. As is possible to observe in Figure 11, an increase of hydrogen concentration leads to *β*-phase that can coexists with the *α*-phase when operating below 300 °C and 20 atm, passing through an unalloyed phase (*α* + *β*), which enhances the gross distortion. In other words, in this miscibility gap, the Pd-clusters related to *β*-phase expands within the *α*-phase, generating mechanical stresses in analogy with the *ball-in-hole* model described above. To relax these stresses, the lattice can react by removing Pd-atoms from the surrounding *α*-phase to the external surface of the metal. Hence, two equilibrium states leading to corresponding equilibrium diagrams can be identified: the *coherent* state and the *incoherent* one. Specifically, coherent solid interfaces are interfaces across which the lattice planes are continuous although not straight. Indeed, in most solids, the lattice lines are elastically strained to keep continuity, and these elastic strains play a crucial role in determining the material properties.

Differently, incoherent solid interfaces are interfaces across which there is no continuity between lattice planes. This situation can be found, for example, in grain boundaries and inclusions in alloys. It must be remarked that a net distinction between both states is actually just ideal, since a distribution of coherent/incoherent phases, named *semi-coherent* state, is in practice achieved [20].

In an ideal coherent equilibrium between two *α*- and *β*-phases, in the *α* + *β* region shown in the bell-shaped diagram, one phase grows up within the parent phase without any diffusive motion of metal atoms to the surface and the lattice is only deformed elastically. As said, the lattice planes are curved but still continuous, and the interface between the phases is characterized by a change both in compositions and in lattice parameters.

The coherent state corresponds to a higher free energy condition than the incoherent state because of the elastic potential energy involved. This chemical potential accumulates until a shift-point from coherency to incoherency is reached. Once this happens, the nucleation of dislocations at the phase interfaces occurs. Ideally, these vacancies should carry metal atoms from the neighborhood to the free surface but, in practice, this transport is never complete, as it is stopped at the grain boundaries. Therefore, the excess free energy of coherent state collapses into plastic deformations characterizing the incoherent state, even though part of that excess energy is not completely removed [20]. The coherence-incoherence dualism is the reason for the well-known hysteresis observed during the definition of coexistence line in the bell-shaped diagram. Coherency stresses as well as their reduction caused by dislocation generation occur in charge/release cycles of hydrogen into/from the lattice whenever more than one phase is generated by the presence of hydrogen [20].

## 4. Conclusions

In the present paper, several thermodynamic aspects related to the hydrogen-metal systems are critically described and discussed. First, the influence of hydrogen-metal interactions on mechanical, structural and electronic properties of several metal alloys is discussed, putting in evidence the detrimental effects provided by a relatively high hydrogen content dissolved in metal lattices.

Within such an analysis, an explicit expression of activity as a function of the hydrogen content in the metal lattice is obtained starting from a complex expression of the hydrogen chemical potential already available in the open literature [22].

The so-obtained activity expression was then used to approach the solution of the solubilization process equilibrium in non-ideal conditions, showing that to be the overall result of two different processes: (a) the molecular hydrogen dissociative adsorption and (b) the solubilization process of the H-atoms adsorbed onto the surface into the metal bulk. For such an analysis, we used a Langmuir-based expression, giving an explicit relationship between surface coverage and hydrogen partial pressure in the gas phase. The obtained expression is shown to extend the original Sieverts’ law, which, differently, is based on Henry’s law. Concerning this aspect, it was highlighted that, in the limit of an infinite dilution system, the obtained non-ideal expression is coincident with Sieverts’ law, as expected for low-concentration systems.

## Figures and Tables

**Table 1:** Lattice constant and corresponding unit cell volume evaluated at 20 °C.

System	*a*, Å	V_cell_, Å^3^	H:Pd Ratio
Palladium	3.890	58.86	-
*α*-phase	3.894	59.05	0.008
*β*-phase	4.025	65.21	0.607

**Table 2:** Critical temperatures *T_c_* and corresponding H:M ratio for selected metals.

Metal	Ref.	*T_c_*	H:M
Vanadium	[46]	170 °C	0.45
Niobium	[47]	140 °C	0.30
Tantalum	[48]	52 °C	0.42

## List of Symbols

*a*	H-activity, -
*a*_1_	Parameter depending on temperature only in Equation (13)
A	Parameter in Equation (6), J mol^−1^
B	Parameter in Equation (6), J mol^−1^
*b*_1_	Parameter in Equation (4), mol^−1^ s^−1^
*E*	Interaction energy, J mol^−1^
*F*	Helmholtz free energy, J mol^−1^
*f*	Fugacity, Pa
*f* ^0^	Reference fugacity, Pa
$\bar{G}$	Partial molar Gibbs free energy, J mol^−1^
$\bar{H}$	Partial molar enthalpy, J mol^−1^
*ħ*	Reduced Planck constant, *h*/2π, J s
$K_{H_{2}}^{}$	Langmuirian hydrogen dissociation constant, Pa^−0.5^
*K_e_*	Equilibrium constant, -
*n*	Number of moles, -
*P*	Pressure, Pa
*r*_1_, *r*_2_	Adsorption and dissolution reactions
*R*	Gas constant, J mol^−1^ K^−1^
$\bar{S}$	Partial molar entropy, J mol^−1^ K^−1^
*U*	Internal Energy, J mol^−1^
*T*	Temperature, K
*V*	Volume, m^3^
*Greek Symbols*	
*φ*	Fugacity coefficient, -
*μ*	Chemical potential, J mol^−1^
*μ*_0_	Chemical potential in the infinite dilution condition, J mol^−1^
*ν*	Poisson ratio, -
*θ*	Surface coverage, -
*ξ*	H-concentration in the metal lattice, -
*ω*	H-atoms local oscillations frequency, mol^−1^ J^−1^ s^−2^
*ω*_0_	H-atoms local oscillations frequency at zero H-content, mol^−1^ J^−1^ s^−2^
*Subscripts, Superscripts and Abbreviations*	
*ads*	Adsorbed phase (on the metal surface)
*conf*	Configurational
*osc*	Oscillatory
*el*	Electronic
*g*	gas phase
H	Referred to Metal–Hydrogen interactions
HH	Referred to Hydrogen–Hydrogen interactions
*ov*	Overall
Perm	Permeate side
*sol*	Solution state (dissolved in the lattice)
*Acronyms*	
BCC	Body-Centered Cubic
BCT	Body-Centered Triclinic
FCC	Face-Centered Cubic
